# Use of Typhoid Vi-Polysaccharide Vaccine as a Vaccine Probe to Delineate Clinical Criteria for Typhoid Fever

**DOI:** 10.4269/ajtmh.19-0968

**Published:** 2020-06-22

**Authors:** Md. Taufiqul Islam, Justin Im, Faisal Ahmmed, Deok Ryun Kim, Ashraful Islam Khan, Khalequ Zaman, Mohammad Ali, Florian Marks, Firdausi Qadri, Jerome H. Kim, John D. Clemens

**Affiliations:** 1 International Centre for Diarrheal Disease Research, Bangladesh, Dhaka, Bangladesh;; 2 International Vaccine Institute, Seoul, Republic of Korea;; 3 Johns Hopkins University, Baltimore, Maryland;; 4 Department of Medicine, University of Cambridge, Cambridge, United Kingdom;; 5 UCLA Fielding School of Public Health, Los Angeles, California;; 6 Korea University College of Medicine, Seoul, South Korea

## Abstract

Blood cultures (BCs) detect an estimated 50% of typhoid fever cases. There is need for validated clinical criteria to define cases that are BC negative, both to help direct empiric antibiotic treatment and to better evaluate the magnitude of protection conferred by typhoid vaccines. To derive and validate a clinical rule for defining BC-negative typhoid fever, we assessed, in a cluster-randomized effectiveness trial of Vi-polysaccharide (ViPS) typhoid vaccine in Kolkata, India, 14,797 episodes of fever lasting at least 3 days during 4 years of comprehensive, BC-based surveillance of 70,865 persons. A recursive partitioning algorithm was used to develop a decision rule to predict BC-proven typhoid cases with a diagnostic specificity of 97–98%. To validate this rule as a definition for BC-negative typhoid fever, we assessed whether the rule defined culture-negative syndromes prevented by ViPS vaccine. In a training subset of individuals, we identified the following two rules: rule 1: patients aged < 15 years with prolonged fever accompanied by a measured body temperature ≥ 100°F, headache, and nausea; rule 2: patients aged ≥ 15 years with prolonged fever accompanied by nausea and palpable liver but without constipation. The adjusted protective efficacy of ViPS against clinical typhoid defined by these rules in persons aged ≥ 2 years in a separate validation subset was 33% (95% CI: 4–53%). We have defined and validated a clinical rule for predicting BC-negative typhoid fever using a novel vaccine probe approach. If validated in other settings, this rule may be useful to guide clinical care and to enhance typhoid vaccine evaluations.

## INTRODUCTION

Typhoid fever is a poverty-related disease, with a high disease burden estimated at 14.3 million cases and approximately 200,000 deaths per year globally^1^ found disproportionately in low- and middle-income countries.^2,3^ Unfortunately, the standard diagnostic test, culture of blood, is only about 50% sensitive in detecting typhoid fever,^4–7^ and there are neither reliable point-of-care diagnostic tests nor well-validated rules for defining blood culture (BC)-negative typhoid using combinations of presenting clinical signs and symptoms to guide empiric antibiotic therapy nor to permit complete evaluation of the preventive impact of typhoid vaccines.^8,9^ The need for more selective targeting of patients for antibiotic therapy is underscored by growing antibiotic resistance and the need for antibiotic stewardship.^10–12^ Similarly, the need for more comprehensive assessments of prevented typhoid cases is highlighted by the advent of improved typhoid vaccines now under clinical evaluation.^13–17^

One of the challenges confronting development of non–BC-based criteria for typhoid fever is the absence of gold standard diagnostic tests for such cases. For several other invasive bacterial diseases, including invasive *Haemophilus influenzae* type b and *Streptococcus pneumoniae*, vaccine probe studies have been used to define pathogen-attributable disease syndromes in which cultures of normally sterile fluids are negative.^18–22^ The probe design, which relies on detecting vaccine efficacy against these culture-negative clinical syndromes, can produce an estimate of the aetiological fraction of a disease syndrome because of the pathogen targeted by vaccine, effectively overcoming challenges in diagnostic insensitivity.^22^ However, this approach only works when a clinical syndrome can be defined for which the proportion of culture-negative disease caused by the target pathogen is sufficiently high. If low, measured vaccine protection will be “washed out” by cases not caused by the target pathogen.

In an earlier cluster-randomized trial (CRT) of typhoid Vi-polysaccharide (ViPS) vaccine, it was possible to detect both direct and herd protection of Vi against BC-proven typhoid.^23^ However, it was not possible to detect significant Vi protection, either direct or herd, against BC-negative cases presenting with fever of at least 3 days duration, suggesting that we needed more specific clinical criteria for defining the target syndrome than prolonged fever per se. However, we lacked these clinical criteria. In this article, we present a novel approach for defining clinical criteria for culture-negative, clinical typhoid leveraging a vaccine probe analysis within the earlier CRT of the ViPS vaccine.

## METHODS AND MATERIALS

### General approach.

We reanalyzed a CRT of ViPS vaccine launched in 2004, as well as 2 years of antecedent typhoid surveillance in the vaccine trial population, in urban slums in Kolkata, India.^23^ To derive an empirically validated clinical rule for BC-negative, clinical typhoid fever, we required that three conditions be met: 1) the clinical features for the rule should also discriminate between episodes of prolonged fever that are BC positive for *S.* Typhi and those that are negative for *S.* Typhi, 2) the diagnostic specificity of the rule should yield a number of BC-negative clinical typhoid episodes approximately equal to the number of BC-positive episodes in the same population (because BCs are known to be about 50% sensitive in detected typhoid fever),^24–28^ and 3) a rule meeting the first two conditions should identify BC-negative episodes against which there was detectable vaccine efficacy by ViPS vaccine in the CRT, thereby using ViPS vaccine as a probe. To derive a clinical rule with the desired level of diagnostic specificity, we used recursive partitioning, a classification algorithm that considers candidate individual variables for the rule conjointly to arrive at a rule with desired levels of specificity and sensitivity.^29^ This approach has several advantages over other machine learning classification algorithms, namely, that it produces an easily visualized decision tree allowing for simplistic interpretation of subject classification.

### The ViPS vaccine trial in Kolkata.

The study was conducted in an urban slum site in Kolkata city, the capital of West Bengal, India. The Kolkata Municipal Corporation area had an estimated population of six million at the time of the trial. A census of 66,458 people was conducted 2 years before the onset of the trial. Individual- and household-level demographic and socioeconomic data were subsequently updated at regular intervals in a demographic surveillance system that continued through the vaccine trial. A close-out census was carried out at the end of surveillance, 2 years after vaccination. Typhoid fever surveillance was carried out in the study population during a 2-year lead-in period before vaccination and continued for 2 years following vaccination. Subjects from the study area who presented at one of five study clinics with a history of fever for at least 3 days were enrolled by a study physician. Subjects’ clinical histories and physical findings were systematically captured during enrollment. After obtaining verbal informed consent, a 5-mL blood specimen was collected by venipuncture for microbiological culture using an automated system (Becton Dickinson, Franklin Lakes, NJ) and bacterial identification through conventional biochemical and serologic methods.^30^ The CRT was initiated in 2004. Residents aged 2 years and older in 80 clusters were randomly allocated at a one-to-one ratio to Vi vaccine (Typherix, GlaxoSmithKline, Brentford, United Kingdom) or inactivated hepatitis A (HepA) vaccine (Havrix, GlaxoSmithKline). A total of 18,869 participants received Vi vaccine, and 18,804 received HepA vaccine. After 2 years of surveillance, the total Vi-vaccine protection against typhoid fever (comparing the incidence of typhoid in Vi vaccinees versus HepA vaccinees) was 61%; indirect protection (comparing the incidence of typhoid in non-vaccinees in the Vi clusters versus non-vaccinees in the HepA clusters) and overall protection (comparing the incidence of typhoid in all members of the Vi clusters versus all members of the HepA clusters) were 44% and 57%, respectively.^23^

### Definition and assembly of prolonged fever episodes for analysis.

**Figure 1.**
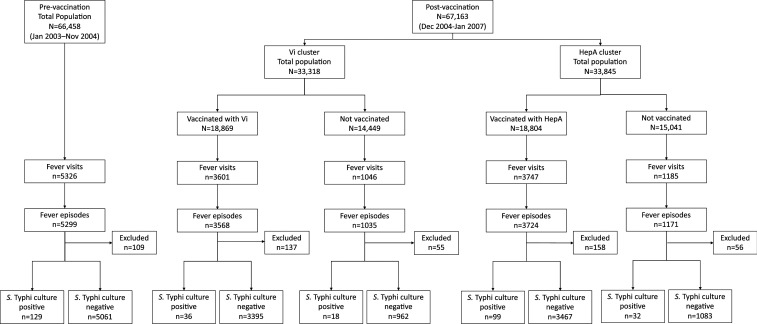
Flow diagram showing culture-confirmed typhoid fever in Vi and HepA clusters.

We assembled all prolonged febrile episodes from the surveillance leading up to and during the cluster-randomized trial. A febrile visit was defined as an individual visit by a study area resident to a study clinic presenting with a history of fever for at least 3 days. A febrile episode was defined as all component febrile visits in which the onset of symptoms for a subsequent visit was less than 14 days from the date of discharge for the previous visit. A BC-proven typhoid episode was considered to be a febrile episode in which *Salmonella enterica* serovar Typhi (*S.* Typhi) was isolated from at least one BC. For this analysis, where multiple fever visits comprised a febrile episode, we considered the date of onset of illness to be the date of onset of symptoms for the first component visit of the episode. From these episodes, analysis included individuals for whom data for all clinical parameters under consideration (vide infra) were available (Figure 1).

### Statistical analysis.

We used a machine learning algorithm to create a decision tree constructed via binary recursive partitioning.^31^ The decision tree was designed to predict whether a febrile episode was culture-positive typhoid fever, using 22 clinical signs and symptoms and epidemiological characteristics recorded at the time of initial presentation for care for the episode. Each sign and symptom was first converted into a dichotomous variable. For the machine learning modeling, we selected as candidate variables only those that had an association with typhoid with *P*-value ≤ 0.25 in bivariate logistic regression analyses.

Because predictive rules may overestimate predictive performance when tested on the data used to derive the rules, we split all individuals who resided in the study area during the 4 years of follow-up, at random, into two approximately equal-sized groups, for development (training dataset) and validation (validation dataset) of the clinical predictive rule, respectively. For development and testing of the rule, we considered all febrile episodes of all patients presenting with at least 3 days of fever in the training and validation groups for whom complete clinical data were available. We initially ran the recursive partitioning algorithm for candidate clinical predictive variables in the training set episodes, specifying an alternative loss function that penalized a false-negative prediction 10 times more heavily than a false-positive prediction. Because of unbalanced class data, we set the minimum number of observations as 30 for the nonterminal nodes of the decision tree and 10 for the terminal nodes of the decision tree. For cross-validation, the training set was randomly partitioned into 10 parts, one part was used in estimating cross-validation error.^32^

For these analyses of the training dataset, we constructed trees at several different levels, choosing the tree with the lowest level, and thus the simplest clinical algorithm, that still met our three performance criteria (vide supra). To find the optimal tree in the training set, the model was pruned by the minimal complexity parameter corresponding to minimum error with at least five tree rules. We constructed a receiver operating characteristic (ROC) curve for the selected rule, evaluating the area under the ROC curve as described elsewhere,^33,34^ and also used the curve to identify a cutoff probability that corresponded to the highest sensitivity for cutoffs, giving 97–98% specificity. To measure the protective efficacy (PE) of ViPS vaccine against culture-negative typhoid, we contrasted ViPS vaccine recipients with HepA vaccine recipients for the occurrence of all episodes of culture-negative fever meeting the criteria of the clinical prediction rule, using the Andersen–Gill (AG) generalized Cox proportional hazard regression model after verifying that the proportionality assumptions were fulfilled.^35^ The model was adjusted for age of the individual and living in a household that used a designated place for waste disposal. The hazard ratio (HR) for culture-negative typhoid fever was estimated by exponentiation of the coefficient of receipt of ViPS in the model, and the 95% CI for the HR was estimated using a robust sandwich method. The protective effectiveness (PE) of ViPS vaccination against BC-negative typhoid fever defined by our clinical rule was estimated as (1-HR) × 100%. We considered *P* < 0.05 (two-tailed) as the margin of statistical significance. The analysis was performed using “rpart” package for decision tree modeling, “caTools” for random selection, “rpart.plot” package for tree plotting, “pROC” packages for ROC curve,^36^ “survival” for AG Cox model,^37^ and “dplyr” package for data management under R-Studio.^38^ Once having conducted these developmental analyses in the training dataset, we assessed the sensitivity and specificity of the rule in predicting BC-positive typhoid fever and in defining a BC-negative syndrome against which ViPS protected in the validation dataset.

## RESULTS

From March 2003 to January 2007, a total population of 70,865 individuals was included in the surveillance. During vaccination, which took place between November 2004 and December 2004, 67,163 age-eligible individuals were randomized to receive either ViPS vaccine (18,869 vaccinated) or HepA vaccine (18,804 vaccinated) (Figure 1). Total protection by ViPS was 61% (95% CI: 41–75; *P* < 0.001); complete results of the vaccine trial have been reported previously.^23^ A total of 14,905 febrile visits, comprising 14,797 fever episodes, in 11,255 individuals were observed between March 19, 2003 and January 28, 2007, from which 323 BC-proven typhoid cases were recorded. For our analysis, 515 fever episodes, including nine typhoid cases, were excluded due to incompatibility with fever criterion (i.e., fever duration less than 3 days) or incomplete clinical metadata (Figure 1). We included 14,282 fever episodes and 314 BC-proven typhoid cases in the analysis. From bivariate logistic regression analysis, 17 of 22 recorded clinical and epidemiological features had a significant association (*P*-value ≤ 0.25) with confirmed typhoid. These include age < 15 years, typhoid season (April to June and September to November), high temperature (≥ 100°F by axillary measurement), abdominal pain, constipation, dehydration, diarrhea, abdominal distension, drowsiness, headache, nausea, palpable liver, palpable spleen, abdominal tenderness, heart rate > 80/minute, thirst, and vomiting (Table 1). Blood culture–confirmed typhoid was not observed among individuals exhibiting abdominal peritoneal signs or seizure.

**Table 1:** Bivariate relationships* between candidate clinical and epidemiological features and typhoid fever

Feature	Culture-negative fever episode (*n* = 13,968), *n* (%)	Culture-positive typhoid fever episode (*n* = 314), *n* (%)	*P*-value
Age (< 15 years)†	4,742 (33.9)	191 (60.8)	< 0.001
Gender (male)	7,180 (52.1)	159 (51.8)	0.919
Typhoid season‡	7,573 (54.2)	201 (64.0)	0.001
Fever duration (> 7 days)§	1,238 (8.9)	28 (8.9)	0.973
High temperature (≥ 100°F)‖	7,012 (50.2)	205 (65.3)	< 0.001
Abdominal pain	1,711 (12.2)	71 (22.6)	< 0.001
Constipation	1,752 (12.5)	47 (15.0)	0.201
Dehydration	13,134 (94)	284 (90.4)	0.009
Diarrhea	374 (2.7)	12 (3.8)	0.219
Abdominal distension	709 (5.1)	24 (7.6)	0.043
Drowsiness	110 (0.8)	8 (2.5)	0.001
Headache	8,015 (57.4)	195 (62.1)	0.095
Ileus sign	5 (0)	0 (0)	0.967
Nausea	3,312 (23.7)	95 (30.3)	0.007
Palpable liver	577 (4.1)	22 (7)	0.013
Peritoneal signs	6 (0)	0 (0)	0.964
Seizure	19 (0.1)	0 (0)	0.972
Palpable spleen	267 (1.9)	9 (2.9)	0.228
Abdominal tenderness	1,079 (7.7)	36 (11.5)	0.015
Heart rate (> 80 bpm)	12,054 (86.3)	299 (95.2)	< 0.001
Thirsty	3,455 (24.7)	117 (37.3)	< 0.001
Vomiting	1,341 (9.6)	42 (13.4)	0.026

* Assessed with logistic regression.

† Age was considered at the date of fever onset.

‡ Typhoid seasons defined as April to June and September to November.

§ By reported clinical history.

‖ Axillary, oral, or rectal temperature measurement.

### Assembly of training and validation datasets.

In the training dataset, 7,195 febrile episodes in 5,462 individuals were randomly selected. Among these episodes, 1,748 and 1,799 episodes were from ViPS vaccine and HepA vaccine recipients, respectively, occurred after vaccination. The rest of the episodes were from the nonparticipants in the trial. Similarly, 7,087 febrile episodes from the 5,462 individuals were randomly selected for the validation set, of which 1,683 and 1,767 episodes were from ViPS vaccine and HepA vaccine recipients, respectively, occurred after vaccination (Table 2).

**Table 2:** Population assembly for training and validation sets

			Population	Full study period		Before vaccination		After vaccination	
			*N*	Fever episodes (subjects)	Typhoid cases	Fever episodes	Typhoid cases	Fever episodes	Typhoid
Full set	Before vaccination		3,702	102	4	102	4		
	Vi cluster	Vaccinated	18,869	5,438	91	2,007	55	3,431	36
		Not vaccinated	14,449	1,459	26	479	8	980	18
	HepA cluster	HepA vaccinated	18,804	5,639	147	2,073	48	3,566	99
		Not vaccinated	15,041	1,644	46	529	14	1,115	32
		Total	70,865	14,282 (10,924)	314	5,190	129	9,092	185
Training set	Before vaccination		1,851	56	2	56	2		
	Vi cluster	Vaccinated	9,436	2,752	41	1,004	21	1,748	20
		Not vaccinated	7,197	732	11	217	4	515	7
	HepA cluster	HepA vaccinated	9,404	2,846	77	1,047	31	1,799	46
		Not vaccinated	7,540	809	26	257	11	552	15
		Total	35,428	7,195 (5,462)	157	2,581	69	4,614	88
Validation set	Before vaccination		1,851	46	2	46	2		
	Vi cluster	Vi vaccinated	9,433	2,686	50	1,003	34	1,683	16
		Not vaccinated	7,252	707	13	262	4	465	11
	HepA cluster	HepA vaccinated	9,400	2,793	70	1,026	17	1,767	53
		Not vaccinated	7,501	816	20	272	3	563	17
		Total	35,437	7,087 (5,462)	157	2,609	60	4,478	97

Note: For randomized individuals who did not receive a vaccine, vaccine date was set as the median date of vaccination of vaccinees in the cluster of residence.

### Analysis of training dataset.

**Figure 2.**
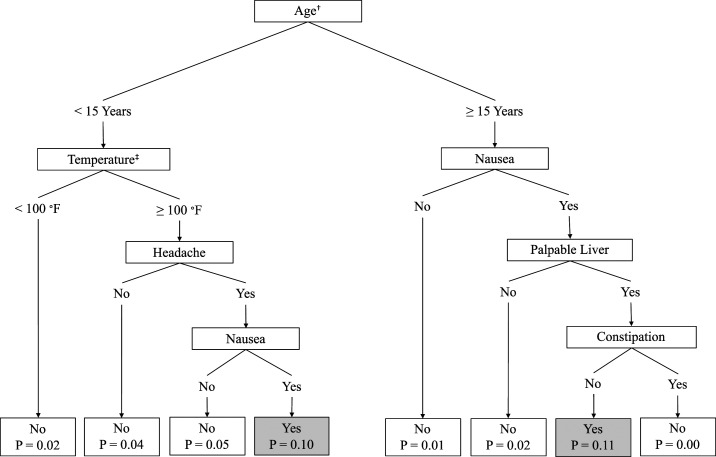
Depth four decision tree specifying clinical and epidemiological criteria for prediction of blood culture–positive typhoid. Note: Prediction (represented by terminal node) cutoff probability (P) is set as 0.09. † Age was considered at date of fever onset. ‡ Axillary, oral, or rectal temperature measurement.

The training set was used to build a training model to predict BC-proven typhoid. The model was tuned by varying cutoff probabilities starting from 0% to 50% incremented by 1% using the grid-search technique,^39^ and the predefined specificity (97–98%) was matched at 6–10% cutoff probabilities. A decision tree with a depth of four was selected as the optimal model because it was able to provide a sufficiently simple rule for clinical diagnosis and yielded specified probability cutoff range of 9%. We derived the following rules for defining BC-proven typhoid in the population (Figure 2).

Rule 1: patients aged < 15 years with high temperature (≥ 100°F), headache, and nausea.

Rule 2: patients aged ≥ 15 years with nausea, palpable liver, and without constipation.

**Figure 3.**
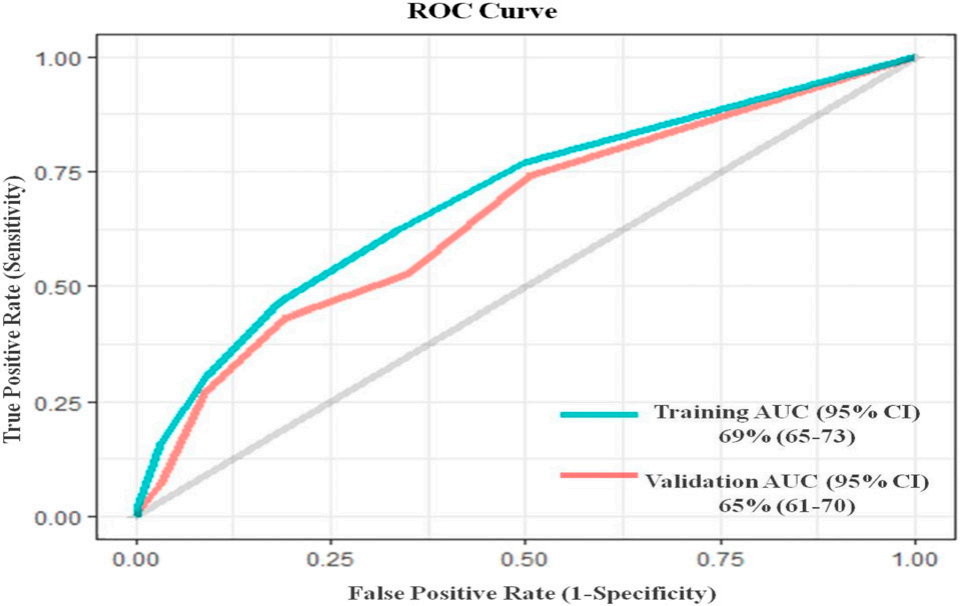
Receiver operating characteristic curve for different cutoff values for defining typhoid in the training and validation datasets. AUC = area under the curve. This figure appears in color at www.ajtmh.org.

Overall, the accuracy of the training model was 95%, with 16% sensitivity and 97% specificity, and the area under curve (AUC) showed significant difference from the 50% random line, measured as 69% (95% CI: 65–73) (Figure 3). When these criteria were used to define BC-negative syndromes, the adjusted PE of the ViPS vaccine was 31% (95% CI: 2–52, *P* = 0.040) for all age-groups (Table 3). Evaluations of the validation dataset yielded similar results. Overall, the accuracy of the validation model was 95%, with 97% specificity. The sensitivity of the model decreased from 16.0% to 7.0%; however, the AUC from the ROC curve remained significant at 65% (95% CI: 61–70) (Figure 3). The vaccine PE of ViPS vaccine against BC-negative syndromes meeting the derived clinical criteria in the validation set was 33% (95% CI: 4–53, *P* = 0.027) for all age-groups. By comparison, the PE of ViPS vaccine against all fever episodes was 0% (95% CI: −7 to 7) and 1% (95% CI: −6 to 8) in the training and validation datasets, respectively (Supplemental Table 1).

**Table 3:** Protective effectiveness of Vi-polysaccharide vaccine against clinical typhoid as defined by rules 1 and 2

	Vi vaccine group				HepA vaccine group				Protective effectiveness (PE)			*P*-value
	*N*	case*	PY	IR per 10^5^ PY	*N*	case*	PY	IR per 10^5^ PY	Crude PE	*P*-value	Adj. PE	
Training set												
All ages (years)	9,436	51	18,345	278	9,404	77	18,266	422	34 (6, 54)	0.021	31 (2, 52)†	0.040
2–14	2,657	49	5,143	953	2,847	74	5,511	1,343	29 (−2, 51)	0.062	29 (−2, 50)‡	0.063
≥ 15	6,779	2	13,202	15	6,557	3	12,754	24	36 (−285, 89)	0.630	36 (−285, 89)‡	0.630
Validation set												
All ages (years)	9,433	52	18,320	284	9,400	79	18,242	433	34 (7, 54)	0.018	33 (4, 53)†	0.027
2–14	2,722	49	5,284	927	2,832	75	5,486	1,367	32 (3, 53)	0.035	32 (3, 53)‡	0.033
≥ 15	6,711	3	13,035	23	6,568	4	12,756	31	27 (−227, 84)	0.684	27 (−227, 84)‡	0.684

IR = incidence rate; PE= (1−HR) ×100; PY = person-years. Age at date of vaccination.

* Based on clinical typhoid end point, defined as either rule 1 (patients aged < 15 years with high temperature (≥ 100°F), headache, and nausea) or rule 2 (patients aged ≥ 15 years with nausea, palpable liver, and without constipation).

† Protective effectiveness is adjusted for age and individual living in a household using a specific place for waste disposal.

‡ Protective effectiveness is adjusted for individual living in a household using a specific place for waste disposal.

## DISCUSSION

Our analysis demonstrates that it is possible to use clinical, demographic, and epidemiological variables to describe a clinical syndrome for typhoid in a manner which maintains acceptable diagnostic specificity for BC-proven typhoid, yielding a plausible number of clinical typhoid cases in the BC-negative group, and demarcates a group of BC-negative, persistent febrile episodes against which ViPS vaccine protects. We believe that this is the first time that an effective typhoid vaccine has been used as a “probe” to test the validity of clinical criteria for clinical typhoid.

Several studies have investigated the clinical symptoms predictive of proven typhoid fever, although these studies assessed disparate populations and age-groups, include varying ranges of clinical factors, and used different methods to substantiate their findings. Despite this heterogeneity, several notable variables are stated to have a high positive predictive value for culture-confirmed typhoid or to be statistically associated with culture-confirmed typhoid by logistic regression across multiple studies. These clinical variables include fever,^40–44^ relative bradycardia,^45–47^ coated tongue,^46,47^ positive Widal test,^44,47^ vomiting,^40–43^ diarrhea,^40–43^ palpable spleen,^40–44^ and palpable liver.^42–44,48^ A clinical trial of ViPS vaccine in Nepal identified culture-negative patients with clinical suspicion of typhoid fever (defined as prolonged fever of three consecutive days, heart rate ≤ 80 beats per minute, and palpable spleen) for whom ViPS vaccine was 80% efficacious; however, specificity or sensitivity of these criteria was not reported.^49^ When we examined these criteria from the Nepal trial for culture-negative typhoid in our data, we found that there were four episodes in HepA vaccinees versus zero episodes in ViPS recipients. To our knowledge, this is the first study to offer a validated clinical predictor rule to diagnose typhoid using clinical data.

Our findings should be interpreted in light of several important limitations. First, reporting of clinical signs and symptoms may have suffered from errors due to limitations in the accuracies of medical histories provided by patients and the examinations performed by clinicians under hectic outpatient clinic conditions in crowded study facilities in urban slums. However, our findings do provide a realistic picture of diagnostic rules that can be used in such settings, which are common in typhoid-endemic populations in the developing world. Second, we did not consider point-of-care diagnostic laboratory results, as these were not uniformly available at the time of presentation in the clinics. Again, the absence of availability of such data for clinical decision-making at the time of patient presentation may be the rule, not the exception, in typhoid-endemic settings. Third, we did not include any post-presentation clinical information—which would not be applicable to the needs of clinicians making decisions on initial presentation of febrile patients, but might be of interest for other purposes, such as classifying BC-negative typhoid end points for a typhoid vaccine efficacy trial. Fourth, our analysis contained a mix of vaccinated and unvaccinated febrile patients. In future analyses, we will consider the applicability of our clinical prediction rule to these two types of patients separately. Fifth, it is possible that our findings are idiosyncratic to the population and epidemiological setting for the study. Further assessments in disparate populations are required to determine whether clinical predictors might be generalizable from one setting to another. Finally, the level of protective effectiveness observed in the Vi probe analysis in the training and validation sets, although statistically significant and consistent between the two sets, was lower than the 61% protection by Vi against BC-proven typhoid in the Kolkata trial. This could mean that our rules for clinical typhoid were insufficiently specific and that Vi protection is indeed lower against BC-negative typhoid fever, or both.

However, several considerations strengthen our findings. First, the study was a large, prospective, population-based cohort, comprehensively followed and systematically evaluated for febrile episodes and for typhoid using well-validated microbiological methods in experienced clinical laboratories. Second, the accumulated data were sufficient in size to make use of independent cohorts to both derive and test the clinical prediction rule. Moreover, validation of the derived rule was strengthened by demanding high diagnostic specificity and using the results of a vaccine probe analysis. Finally, vaccine protection against all persistent fever episodes was not detected, demonstrating the discriminatory value of our predictive rule.

In the absence of a sensitive typhoid point-of-care diagnostic, a reliable and highly specific clinical rule for predicting typhoid, irrespective of culture positivity, will be useful to guide healthcare workers practicing in endemic, low-resource settings.^50^ With a new series of phase III and IV trials being undertaken in Bangladesh, Nepal, and Malawi to evaluate new generation, Vi-protein conjugate vaccines,^16,51–53^ our vaccine probe approach should be considered in future analyses to confirm and extend our findings.

## Supplemental table

Supplemental materials
