# Supplementary material for: Use of Typhoid Vi-Polysaccharide Vaccine as a Vaccine Probe to Delineate Clinical Criteria for Typhoid Fever
Source: Am J Trop Med Hyg. 2020 Jun 22;103(2):665–71. doi: 10.4269/ajtmh.19-0968 (PMC7410438; doi:10.4269/ajtmh.19-0968)
Supplement: Supplementary file 1 [file tpmd190968.SD1.docx]

Supplement Table 1: Protective effectiveness (PE) of Vi-PS vaccine against all fever episodes

|  | Vi Vaccine Group | | | | Hep.A Vaccine Group | | | | Protective Efficacy (PE) | | | |
| --- | --- | --- | --- | --- | --- | --- | --- | --- | --- | --- | --- | --- |
|  | N | Fever  episodes | PY | IR per  10^5^ PY | N | Fever  episodes | PY | IR per  10^5^ PY | Crude PE | p-value | Adj. PE | p-value |
| Training Set | |  |  |  |  |  |  |  |  |  |  |  |
| All age | 9436 | 1685 | 16961 | 9934 | 9404 | 1709 | 16884 | 10122 | 2 (-5, 8) | 0.584 | 0 (-7, 7)^†^ | 0.933 |
| 2-14 yrs | 2657 | 725 | 4600 | 15762 | 2847 | 764 | 4949 | 15437 | -2 (-13, 8) | 0.702 | -2 (-13, 8)^‡^ | 0.682 |
| 15+ yrs | 6779 | 960 | 12362 | 7766 | 6557 | 945 | 11935 | 7918 | 2 (-7, 10) | 0.661 | 2 (-7, 11)^‡^ | 0.652 |
| Validation Set |  |  |  |  |  |  |  |  |  |  |  |  |
| All age | 9433 | 1641 | 16912 | 9703 | 9400 | 1677 | 16913 | 9915 | 2 (-5, 9) | 0.544 | 1 (-6, 8)^1†^ | 0.752 |
| 2-14 yrs | 2722 | 711 | 4678 | 15200 | 2832 | 732 | 4964 | 14748 | -3 (-14, 7) | 0.573 | -4 (-15, 7)^‡^ | 0.485 |
| 15+ yrs | 6711 | 930 | 12234 | 7602 | 6568 | 945 | 11950 | 7908 | 4 (-5, 12) | 0.398 | 4 (-5, 12)^‡^ | 0.394 |

***Note:*** *Age at date of vaccination*

PY = Person years; IR = Incidence rate; PE = (1-HR) × 100

^†^ Protective effectiveness is adjusted for age and individual living in a household using a specific place for waste disposal

^‡^ Protective effectiveness is adjusted for individual living in a household using a specific place for waste disposal
